# Aerogel Poly(butylene succinate) Biomaterial Substrate for RF and Microwave Applications

**DOI:** 10.1038/srep12868

**Published:** 2015-08-04

**Authors:** M. Habib Ullah, W. N. L. Mahadi, T. A. Latef

**Affiliations:** 1 Dept. of Electrical Engineering, Faculty of Engineering, University of Malaya, 50603 Kuala Lumpur, Malaysia

## Abstract

Polybutylene succinate (PBS) has become a potential candidate, similar to polypropylene (PP) and acrylonitrile butadiene styrene (ABS), for use as an organic plastic material due to its outstanding mechanical properties as well as high thermal deformation characteristics. A new composition of silica aerogel nanoparticles extracted from rice waste with PBS is proposed for use as a dielectric (*ε*_*r*_ *=* *4.5*) substrate for microwave applications. A microstrip patch antenna was fabricated on the proposed dielectric substrate for multi-resonant ultra-wideband (UWB) applications. The performance characteristics of the proposed biomaterial-based antenna were investigated in a far-field measurement environment. The results indicate that the proposed biocompatible material-based antenna covered a bandwidth of 9.4 (2.3–11.7) GHz with stop bands from 5.5 GHz to 5.8 GHz and 7.0 GHz to 8.3 GHz. Peak gains of 9.82 dBi, 7.59 dBi, 8.0 dBi and 7.68 dBi were measured at resonant frequencies of 2.7 GHz, 4.6 GHz, 6.3 GHz and 9.5 GHz, respectively.

Recently, ecological contamination has become a serious threat to the environment due to the extensive use of synthetic non-disposable petroleum-based polymer materials. This issue may be resolved if non-degradable polymers (plastics) are replaced with biodegradable polymers (e.g., polylactic acid (PLA), polyhydroxybutyrate (PHB), soy-based plastics, cellulose polyesters, starch-based bioplastics, and vegetable-oil-derived bioplastics such as poly(trimethylene terephthalate) and biopolyethylene)^1^. Most of the available plastic (polyester) products are composed of petroleum or its allied components, which are not biodegradable and adversely affect biological functions in the environment. Currently, bio-based composite materials have become the subject of extensive studies because they are readily available in nature and ensure green environmental adaptability due to their biodegradability. Poly(butylene succinate) (PBS) has found limited application in packaging, mulching film, shopping and trash bags, and disposable food containers^2^. PBS offers outstanding mechanical properties with excellent thermal stability and a high distortion temperature, which makes it a suitable replacement for conventional nonbiodisposable plastics^2^. However, due to its inherent high crystallinity, the biodegradation rate of PBS is slower than that in other aliphatic polyesters, such as PLA and PHA (polyhydroxyalkanoates)^3^. PBS is typically synthesized via the polycondensation of succinic acid and 1,4-butanediol (BDO)^4^. Previously, succinic acid could be derived only from petrochemical substances. However, succinic acid is available from the bioconversion of renewable resources, such as glucose, xylose, and starch^5^, and 1,4-butanediol can be obtained from the catalytic hydrogenation of succinic acid^6^. Two raw materials that are required for the preparation of PBS can be derived from biomass. Therefore, PBS can be identified as a purely bio-based aliphatic polyester.

PBS is a novel biodegradable aliphatic polyester with good biocompatibility^7^, and its end-degradation products (CO_2_ and H_2_O) are harmless. PBS exhibits not only excellent mechanical characteristics but also excellent biodegradability and biocompatibility^7^,^8^. To the best of our knowledge, no studies on the use of biodegradable PBS as a dielectric substrate material for the development of microwave planar circuitry have been reported. However, some biomaterial substrate materials have been proposed for microwave range applications, such as bioplastic-ceramic-bioplastic sandwich structured material^1^, conductive polymer^9^, and organic liquid crystal polymer^10^. Silica aerogels have received significant attention from academic and industrial researchers due to their exceptional properties and applications in a wide variety of technological areas, such as catalysts and catalytic supports, transparent thermal insulators, membranes, radio luminescent devices, and drug release systems. The aerogel was first introduced by Kistler in 1931^11^ and subsequently developed^12^,^13^,^14^. Organic aerogel is composed of nanoparticles and pores that are portions of the light wavelength. As a highly porous material, the unique microstructure of the aerogel makes it extraordinary among common materials due to its outstanding characteristics. Extensive investigations of highly porous silica aerogels have been conducted, and the high thermal resistance and solar transmission properties of these materials make them suitable for passive solar use^15^. Assimilation of an organic silica aerogel into PBS provides a wide operating bandwidth due to its incredible dielectric and electronic properties. Most of the previous studies have focused on energy absorbers, thermal insulators, synthesized solar cells, clothing, and data storage^16^. Silica aerogel from rice husk can potentially be used as a ceramic material glaze or ceramic pigment, which was reported by Bondioli *et al.* in 2010^17^. Organic aerogel nanoparticles extracted from rice husk have been integrated into PBS biomaterial for use as a promising substitute for petrochemical-based conventional printed circuit board (PCB) substrate materials.

In this study, a new organic silica aerogel nanoparticle synthesized from rice husk incorporated into poly(butylene succinate) biomaterial is proposed as a dielectric substrate for microwave circuitry. The proposed dielectric material can be identified as a biomaterial because all of the raw materials are extracted from a biosource, which is organic, available in nature and environmentally friendly. Previously, we proposed a new ceramic-filled bio-plastic material with high relative permittivity (dielectric constant *ε*_*r*_ *=* *15*) for a dual band antenna^1^. However, high dielectric materials miniaturize the overall size of the antenna at the cost of inadequate bandwidth^18^ and poor radiation performance. The development of biocompatible dielectric substrate materials suitable for wideband/UWB antennas remains challenging. Organic silica aerogels extracted from rice husk incorporated into a biocompatible PBS dielectric material is a potential substitute for traditional non-disposable substrates for microwave PCB. For experimental verification of the hypothesis, a compact microstrip line-fed planar antenna was designed and fabricated on a double-sided copper laminated organic silica aerogel nanoparticle assimilated in a 1.2-mm thick PBS biomaterial substrate. The experimental results indicate that the fabricated antenna can achieve operating bandwidths of 1.8 (2.3–4.1) GHz, 1 (4.4–5.4) GHz, 1 (5.9–6.9) GHz and 3 (8.4–11.4) GHz with average peak gains of 9.35, 7.56, 8.18, 8.7 dBi, respectively. The consistent radiation characteristic of the antenna allows for its use in a wide range of applications.

## Results

### Antenna Design

**Figure 1:**
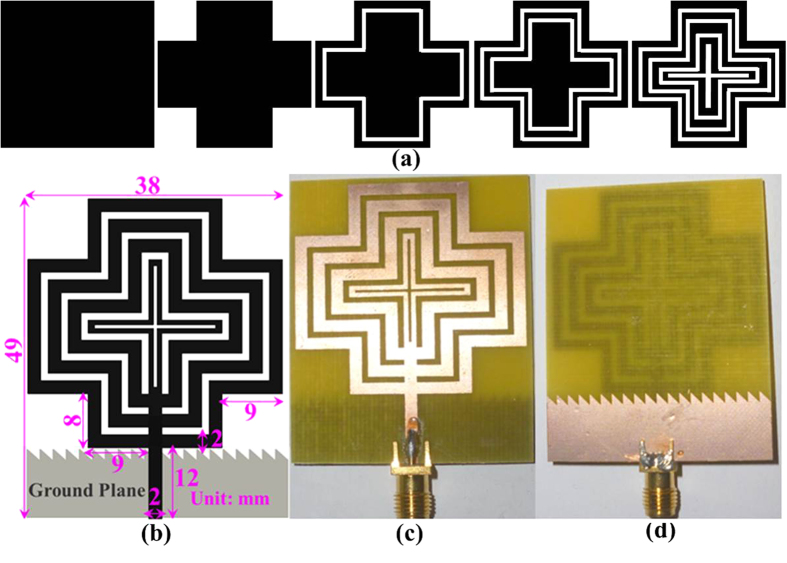
(**a**) Design evolution, (**b**) schematic, (**c**) patch and (**d**) ground plane of the proposed antenna prototype.

**Figure 2:**
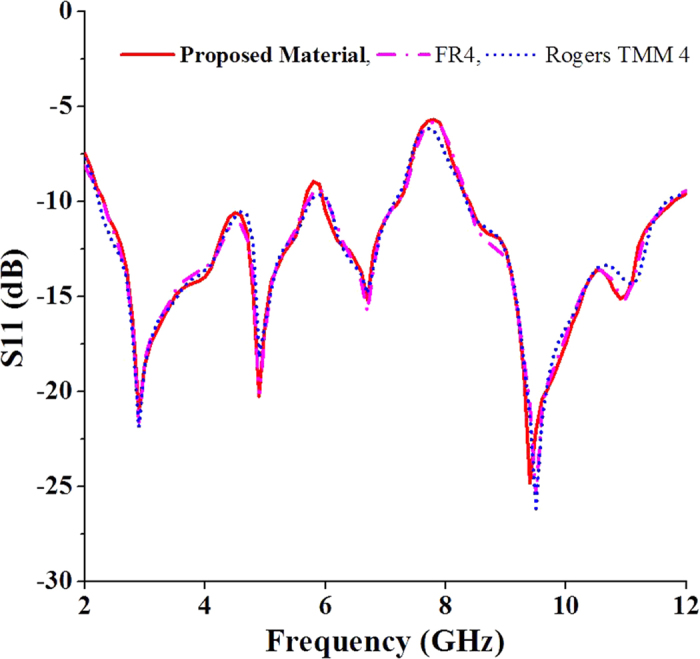
S11 variations of the antenna with different substrate materials.

A new planar microstrip line-fed patch antenna was designed on the proposed dielectric substrate to verify the suitability of the material. The antenna was designed using a widely used full-wave electromagnetic simulator HFSS 14.0 and optimized using Optimetrics from ANSYS Corp. The designed antenna was fabricated on the proposed double-sided copper laminated dialectic material and measured in a standard far field anechoic chamber. A standard 50 Ω Sub-Miniature A (SMA) connector was soldered at the end of the microstrip line to connect the antenna to the co-axial probe for measurement. Figure 1 shows the design evolution, schematic and photograph of the experimental prototype of the antenna. Initially, a simple rectangular radiating patch was chosen for the proposed antenna. However, the resonant frequencies and bandwidths were not achieved. Then, corner tapered, single, dual and triple four-petal ring configurations were tested. The design parameters of the radiating patch was critically optimized to alter the path and direction of the propagating wave to achieve the desired resonant frequencies and wide bandwidth. Figure 2 shows the bandwidth (S11 < −10 dB) of the proposed dielectric substrate-based antenna compared to the commonly used FR4 and Rogers TMM 4 with a dielectric constant of 4.5. Because the dielectric constant of the three substrate materials was 4.5 and the configuration of the antenna is the same, the variations in the S11 profile as a function of the frequency are not significant. The resonant frequencies and bandwidths primarily depend on the antenna size, thickness, dielectric constant and design configurations. High gain and unidirectional radiation patterns were observed by configuring multiple ring slots and increasing the path length of the traveling waves^19^,^20^,^21^. Finally, a four-petal ring-shaped radiating patch was designed and configured to achieve the desired resonant frequencies and operating bandwidth. To further improve the bandwidth, a sawtooth partial ground plane was applied at the bottom of the antenna^22^.

### Material Processing

**Figure 3:**
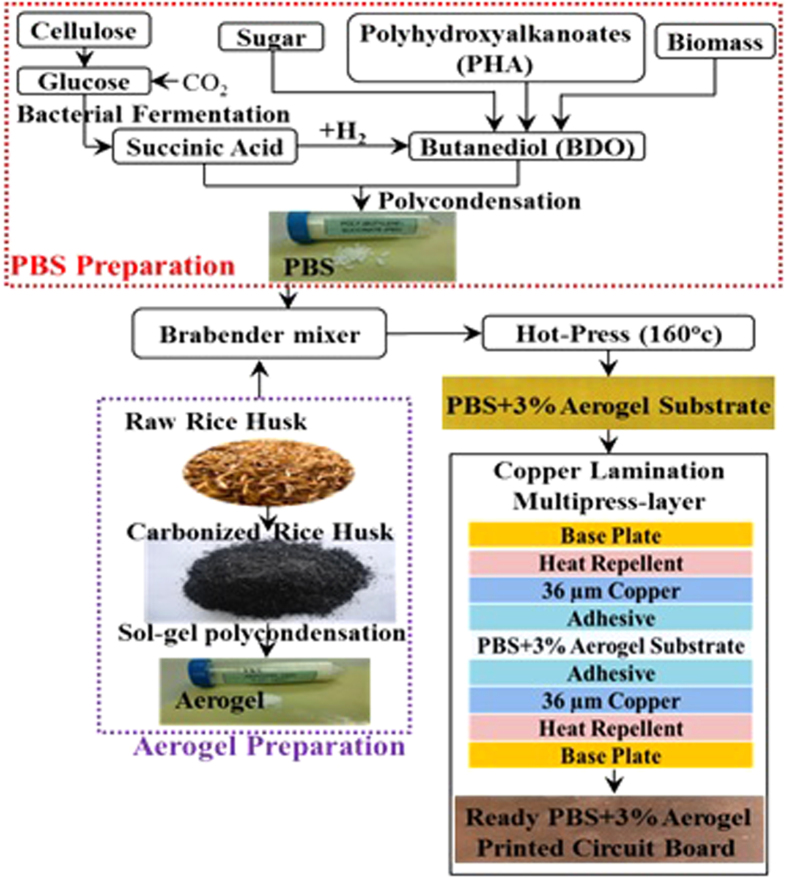
Process flow for the material preparation. (The photograph of this figure was taken by M. Habib Ullah).

The preparation of the proposed silica aerogel-incorporated PBS biomaterial dielectric substrate involved three steps, as shown in Fig. 3. Traditionally, the PBS base material has been prepared from the polycondensation of succinic acid and butanediol (BDO). In general, succinic acid was produced from petroleum or petrochemical-based substances. However, the succinic acid for the proposed material has been derived from the fermentation of microorganisms in renewable feedstocks, such as glucose, starch, and xylose. 1,4-butanediol was processed from biofeedstocks using a Walter Reppe acetylene process^23^. Then, butanediol was synthesized by the hydrogenation of biosuccinic acid and polyhydroxyalkanoates (PHA). PBS was processed by esterification followed by polycondensation of succinic acid and butanediol. Micrometrical high porous aerogels were extracted from rice waste. Initially, rice husk was removed from raw rice in the milling process. Then, the raw rice husk was carbonized by burning at a high temperature (higher than 100 °C), and the silica aerogel was extracted in a dry powder form using the sol-gel polycondensation process with 3% aerogel nanoparticles in powder form mixed with the PBS base material using a brabender from GmbH & Co. mixer. The mixed material was compressed using a hot-press machine at 160 °C to prepare a 1.2 mm thick sheet. The substrate sheet was fragile and cracked once the ratio of the aerogel was increased from 3% to 5% to improve the dielectric constant (*ε*_*r*_).

### Dielectric Testing

The dielectric properties of the prepared substrate material were measured in a Novocontrol (Alpha-A) impedance analyzer that operates up to 20 MHz at room temperature. A computer equipped with user software (WinDETA) was connected to the Alpha-A analyzer mainframe using a GPIB (General Purpose Interface Bus) IEEE488 cable. The test sample was placed in the Zero Gradient Synchrotron (ZGS) sample cell test interface that was also connected to the mainframe analyzer. The impedance converter was directly placed over the sample and connected by an electrode that is combined with the ZGS active sample cell test interface. The impedance of the sample cell was assessed and converted to complex permittivity and/or conductivity by the ZGS active sample cell test interface for the Alpha-A mainframe followed by the conversion of the sample current to two voltages.

**Figure 4:**
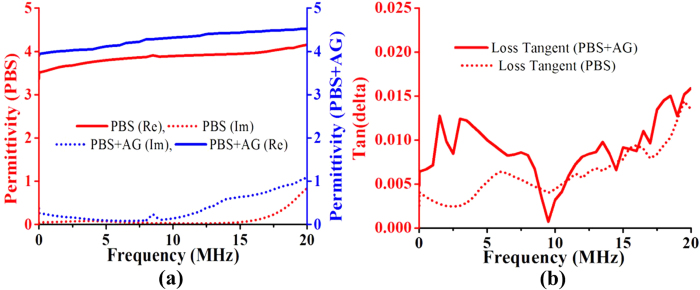
Measured dielectric characteristics of the proposed aerogel integrated PBS and PBS alone. (**a**) permittivity and (**b**) loss tangent.

**Figure 5:**
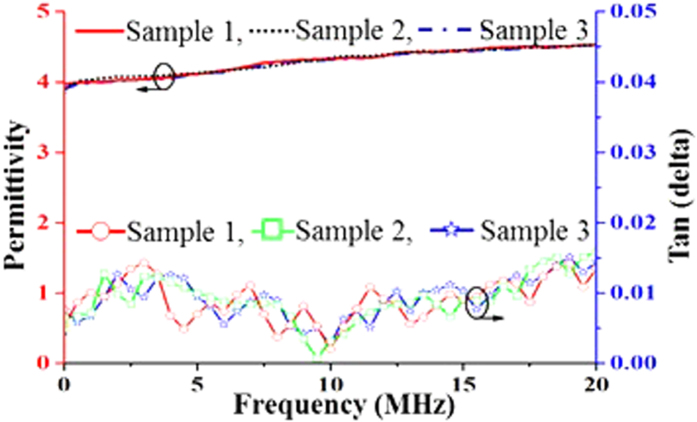
Permittivity and loss tangent of the three proposed dielectric material samples.

Figure 4 shows the dielectric characteristics of the proposed material sample. The permittivity of the proposed aerogel-incorporated PBS is shown in Fig. 4(a). However, due to the limitation of the dielectric testing equipment, the sample was measured in a frequency range of 10 Hz to 20 MHz. At the highest frequency, the real value of permittivity is nearly consistent, and the dielectric constant is considered to be 4.5 (±0.25). Tangent loss of the proposed material is shown in Fig. 4(b), and the tangent loss of the sample is as low as the acceptable label (*tanδ < 0.02*). The permittivity and loss tangent of the three samples of the proposed dielectric material are shown in Fig. 5. Because the size of the three samples and the method of testing were the same, the variations in the permittivity and loss tangent are very low. The fixed diameter of the test sample was 3 cm, which is the same as the test sample panel size of the impedance analyzer.

### Experimental Analysis

**Figure 6:**
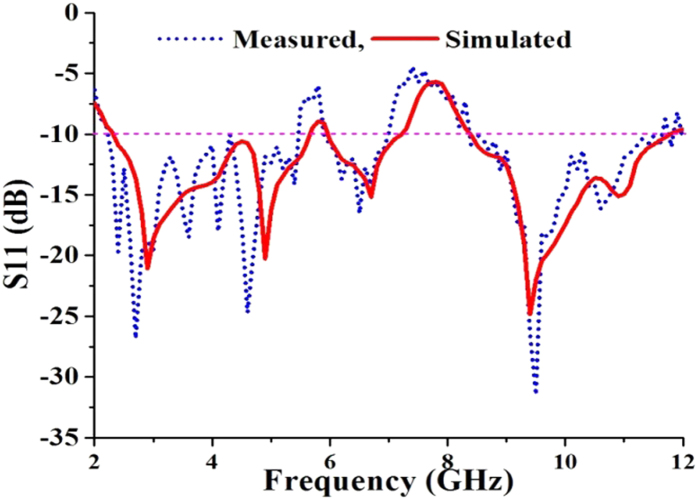
Measured and simulated reflection coefficient (S11) of the antenna.

The reflection coefficient (S11) of the proposed antenna was measured using an Agilent Vector Network Analyzer (PNA E8362C), as shown in Fig. 6. Fair agreement between the numerical simulation and the measured result was observed. The measured results demonstrate that the proposed antenna covers the entire bandwidth (S11 < −10 dB) from 2.3 GHz to 11.7 GHz with a rejected band from 5.5 GHz to 5.8 GHz and 7.0 GHz to 8.3 GHz. Creating a sawtooth-shaped partial ground plane is an effective technique^24^ that was applied to widen the operating bandwidth of the proposed antenna.

**Figure 7:**
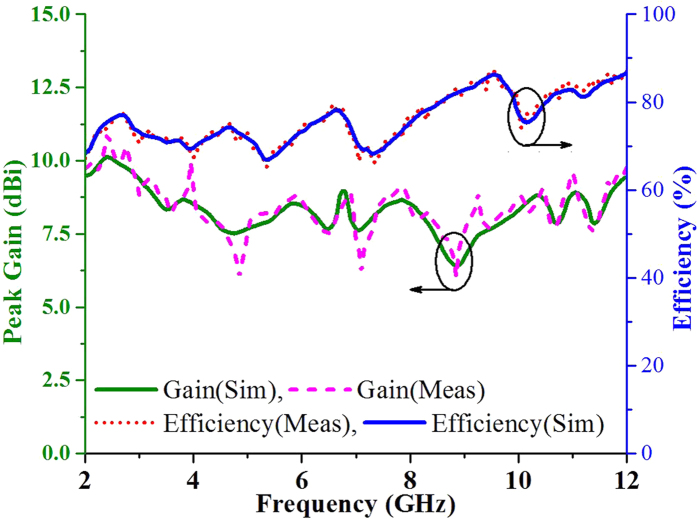
Efficiency and gain as a function of the frequency of the antenna.

**Figure 8:**
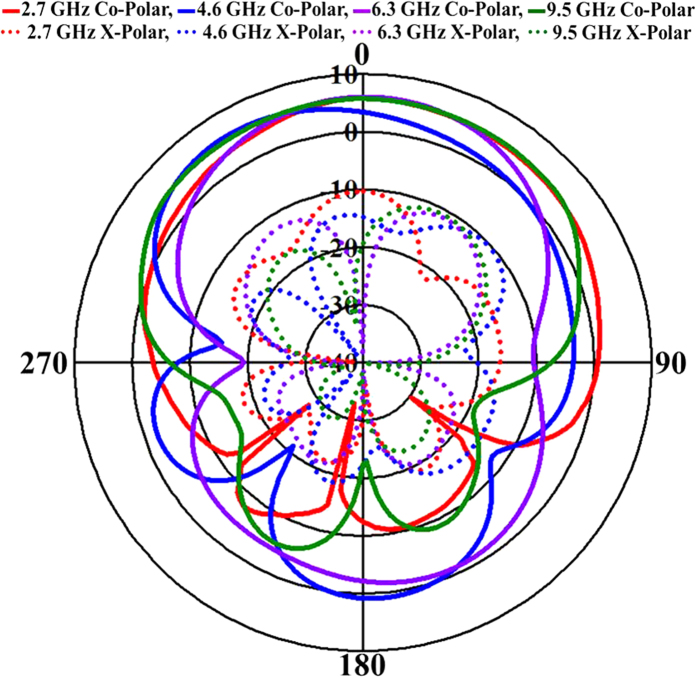
Measured radiation pattern of the proposed antenna at four resonant frequencies.

The simulated and measured peak gain and efficiency over the frequency range of the antenna are shown in Fig. 7. The measured maximum peak gains of 10.88 dBi, 8.92 dBi and 9.59 dBi were realized over the operating bands (2.3 GHz–5.4 GHz, 5.9 GHz–6.9 GHz and 8.4 GHz–11.6 GHz, respectively). The peak gain of the proposed antenna significantly improved due to an increase in the traveling path of the incident wave over the radiating patch. The peak gain of the antenna was measured using a three-antenna gain measurement system with two identical horn antennas^1^. The proposed antenna prototype achieved an average efficiency of more than 75% over the entire frequency band, as shown in Fig. 7. The measured nearly stable and directional radiation patterns of the proposed antenna at resonant frequencies of 2.7 GHz, 4.6 GHz, 6.3 GHz and 9.5 GHz are shown in Fig. 8. A 3-dB beam width was observed from the measured radiation patterns at 188°, 148°, 120° and 166° for four resonant frequencies. Little undesirable backward radiation was observed due to a reduction in the ground plane. Backward radiation can be reduced at the cost of a larger ground plane, which can lead to bandwidth degradation. As expected, the effects of cross polarization are low compared to those of co-polarization. The main features of the proposed antenna include the use of the organic aerogel nanoparticle-assimilated PBS dielectric substrate and the higher performance characteristics, such as wide bandwidth, high gain and stable radiation.

## Discussion

Several biopolymer-based dielectric substrates have been proposed for use in RF and microwave circuitry^1^,^10^,^25^. Due to their fragility, unstable dielectric properties and complicated processing procedures, biopolymer-based dielectric materials remain unpopular for electric circuits compared to petroleum-based materials. In addition, the production, use and subsequent waste of bio-incompatible petrochemical-based electronic components have a detrimental environmental impact. Therefore, bio-sourced dielectric substrate-based electronic components that can reduce the ecological impact and be easily disposed after use are becoming increasingly important.

In conclusion, a new organic aerogel extracted from rice husk and incorporated into PBS has been proposed as a dielectric substrate material for RF and microwave applications. The raw materials of the proposed substrate are extracted from organic biocompatible sources. The preparation and testing procedures have been carried out in a standard environment for laboratory-based research. To confirm the functionality, an antenna was designed, and a prototype using the proposed dielectric material substrate was constructed. The measured results indicate that the antenna achieved operating bandwidths of 1.8 (2.3–4.1) GHz, 1 (4.4–5.4) GHz, 1 (5.9–6.9) GHz and 3 (8.4–11.4) GHz with maximum peak gains of 10.88 dBi, 8.92 dBi and 9.59 dBi, respectively. The key feature of the proposed organic dielectric substrate based-antenna is the use of a porous aerogel from rice husk integrated into PBS for RF and microwave devices. This aerogel can be further extended to the design of other components, such as filters and metamaterials, and the dielectric constant can be further increased using a new composition.

## Additional Information

**How to cite this article**: Habib Ullah, M. *et al.* Aerogel Poly(butylene succinate) Biomaterial Substrate for RF and Microwave Applications. *Sci. Rep.* **5**, 12868; doi: 10.1038/srep12868 (2015).
